# Species Diversity and Community Composition of Macrofungi in the Dongling Mountains, Western Beijing, China

**DOI:** 10.3390/jof11020155

**Published:** 2025-02-18

**Authors:** Jia-Mao Huang, Tai-Min Xu, Wen Zhao, Reyila Mumin, Long Zeng, Yi-Fei Sun, Bao-Kai Cui

**Affiliations:** State Key Laboratory of Efficient Production of Forest Resources, School of Ecology and Nature Conservation, Beijing Forestry University, Beijing 100083, China; yellowhuanghjm@163.com (J.-M.H.); fungitaiminx@163.com (T.-M.X.); zhaowendrlw@163.com (W.Z.); ramilla@163.com (R.M.); zenglong123123@163.com (L.Z.)

**Keywords:** macrofungi, species diversity, community composition, vegetation type, elevation

## Abstract

Macrofungi are a group of fungi with important ecological functions and economic value in forest ecosystems. In this study, the species diversity and community composition of macrofungi in different areas, vegetation types, and elevation gradients from the Lingshan Natural Scenic Area and Xiaolongmen National Forest Park of the Dongling Mountains, Western Beijing were investigated. A total of 1379 specimens were collected. All specimens were identified by morphological and molecular methods, resulting in the identification of 311 species belonging to two phyla, four classes, 18 orders, 74 families, and 147 genera. The alpha diversity analysis indicated that the alpha diversity was significantly different among different vegetation types. The *Betula* spp. and *Populus davidiana* of the Lingshan Natural Scenic Area, and *Quercus liaotungensis* of Xiaolongmen National Forest Park had higher macrofungal alpha diversity. The difference in alpha diversity between the two areas was not significant. The principal coordinate analysis indicated a significant difference in macrofungal community composition among different vegetation types. The fungal community composition of the two areas was also significantly different. The fungal species richness and unique species tended to increase and then decrease with increasing elevation. The species composition of neighboring elevation gradients was more similar. The macrofungal species richness and the number of unique species were not significantly affected by elevation gradient. The vegetation types with higher fungal alpha diversity in the Dongling Mountains were *Betula* spp., *Populus davidiana*, and *Quercus liaotungensis*, and there were abundant fungal species in deciduous broad-leaved mixed forests. Macrofungal diversity and community composition was significantly affected by vegetation type. To better protect the macrofungi, the protection of these four vegetation types in the Dongling Mountains should be strengthened in the future.

## 1. Introduction

Macrofungi are an important component of forest ecosystems [1,2]. Ectomycorrhizal fungi not only promote nutrient cycling in forest ecosystems, but also promote host plants to absorb nutrients, such as nitrogen and phosphorus [3,4]. Moreover, ectomycorrhizal fungi maintained the primary productivity of forest ecosystems [3,4], and protected plant root systems from heavy metal pollution, pathogens, and diseases [5,6]. As active decomposers, saprophytic fungi can degrade lignin, cellulose, and hemicellulose to contribute toward nutrient cycling [7]. Pathogenic fungi impact the productivity, species diversity, and composition of ecosystems [8]. Macrofungi contain polysaccharides, saponin, triterpene, and other components that make them highly nutritious [9]. Additionally, macrofungi have medical functions, such as anti-oxidant, anti-microbial, anti-diabetic, and antitumor functions [10]. In addition, the sensitivity of macrofungi to climate variables makes macrofungi bioindicators of climate change [11]. The high adaptability and survival rates of macrofungi make them widely distributed in environments, such as forests, shrublands, grasslands, and urban regions [12,13].

Macrofungal diversity is influenced by biotic and abiotic factors [14]. The change in forests due to natural succession and anthropogenic disturbance affected macrofungal diversity, functional structure, and community composition [15,16]. The macrofungal distribution was affected by slope aspect [17], microhabitat [18], tree species [19], and human footprint [19]. The previous studies have indicated that abiotic factors, such as substrate [20], precipitation [21], temperature [21], humidity [22], latitude [23], and so on, play key roles in macrofungal diversity and community composition. Wood-decaying fungi were influenced by tree species, the mean volume of logs, and the decomposition stage of deadwood [24,25,26]. Saprophytic fungi were influenced by the volume of deadwood and soil pH [27], tree species [18,28], and temperature [29]. Ectomycorrhizal fungi were influenced by temperature and humidity [30], soil nitrogen deposition [31], tree diversity [32,33], and seasonality and management history [34].

So far, there are still many unknown macrofungi species [35], and there is increasing interest in how to conserve macrofungal diversity [28,36]. The survey sites were located in the Dongling Mountains, Beijing, China, with one of the well-preserved warm-temperate deciduous broad-leaved forests. The Dongling Mountains are part of the Taihang Mountains [37], and have diverse vegetation types and a large elevation drop [38], which makes it possible to study macrofungal diversity and community composition in different vegetation types, areas, and elevations under the same climatic conditions. The macrofungal diversity and community compositions were investigated based on samples from five elevation gradients and seven vegetation types in two areas of the Dongling Mountains.

## 2. Materials and Methods

### 2.1. Study Sites

The study sites were located in the Dongling Mountains (39°48′ N–40°02′ N, 115°24′ E–115°36′ E), Beijing, with 1000–2303 m elevation. The Dongling Mountains mainly include the Lingshan Natural Scenic Area and Xiaolongmen National Forest Park [39]. This region was characterized by a warm-temperate semi-humid continental monsoon climate [40]. The average annual temperature ranged from 5 to 10 °C, the average annual precipitation was 550–650 mm, and nearly 80% of the precipitation occurred from June to August [40]. It is worth noting that the Dongling Mountains suffered from severe rainstorms from 2023 to 2024, especially in the Lingshan Natural Science Area. As the elevation decreases, the vegetation types in sequence are subalpine meadow, *Larix gmelinii*, *Populus davidiana*, *Betula* spp., *Quercus liaotungensis*, and *Pinus tabulaeformis*, which is also interspersed with mixed deciduous and broad-leaved forests, and mixed coniferous and broad-leaved forests [37,41,42].

### 2.2. Sample Plot Setting and Sporocarp Sampling

**Figure 1:**
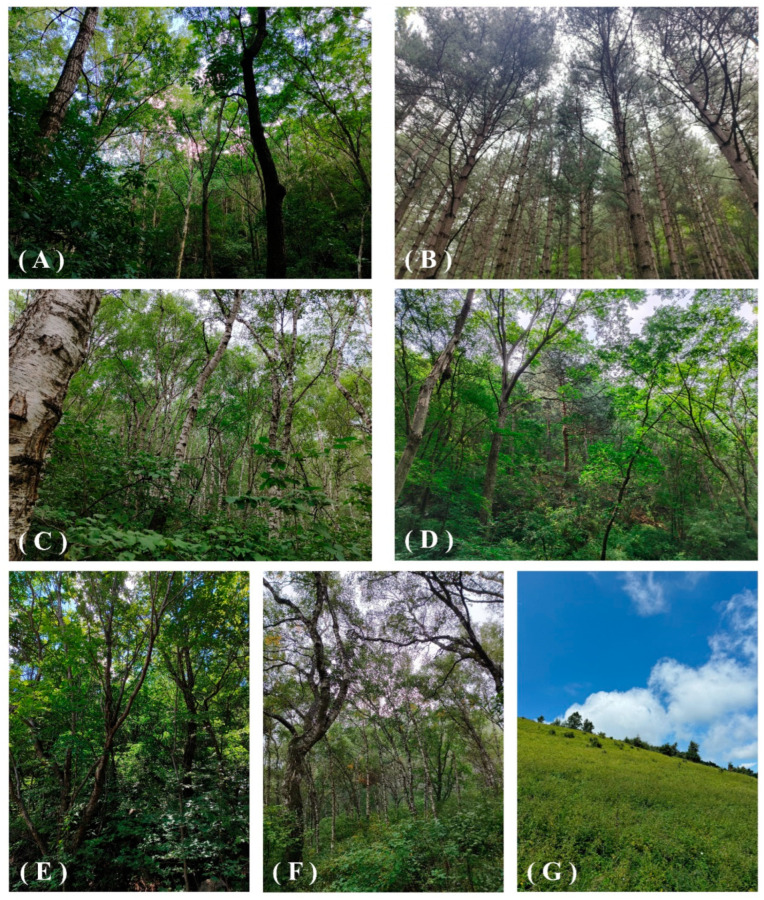
Vegetation types in the Dongling Mountains. (**A**) QLF. (**B**) PTF. (**C**) BSF. (**D**) SUM. (**E**) DBF. (**F**) PDF. (**G**) MBF.

Two areas in the Dongling Mountains with different vegetation types, elevations, and management styles were selected as the study areas, which were the Lingshan Natural Scenic Area and Xiaolongmen National Forest Park. The Dongling Mountains were divided into five elevation gradients according to a 200 m change as an elevation gradient within the range of 1000–2000 m above sea level. Seven vegetation types in the Dongling Mountains were selected to assess macrofungal diversity, including *Quercus liaotungensis* (QLF), *Populus davidiana* (PDF), *Betula* spp. (BSF), *Pinus tabulaeformis* (PTF), subalpine meadow (SUM), deciduous broad-leaved mixed forests (DBF), and mixed coniferous and broad-leaved forests (MBF) (Figure 1). The seven vegetation types belong to two areas, DBF, BSF, DBF, SUM in the Lingshan Natural Scenic Area (LS), and QLF, MBF, PTF in Xiaolongmen National Forest Park (XLM). Three sample plots (20 × 20 m) were set up under each vegetation type over 2023–2024; each sample plot was spaced at least 50 m apart. The macrofungi growing on the ground and trees were collected randomly in each sample plot over 2023–2024 in the Dongling Mountains. The main collecting seasons were summer and autumn (from July to September).

### 2.3. Species Identification

Morphological observations and molecular sequences were used to identify the collected macrofungal specimens. The dried specimens were stained in 5% potassium hydroxide (KOH), Melzer’s reagent, and Cotton Blue; they were then examined with a Nikon Eclipse E 80i microscope (Nikon, Tokyo, Japan) at magnifications up to 1000×. All measurements were taken from the sections mounted in Cotton Blue. The macrofungal features, including the pileus, lamellae, stipe, hyphae, and spores, were used to contrast with referred books and the literature [43,44,45].

The internal transcribed spacer regions (ITS) of the rDNA region were generally used as the DNA barcode region for identifying macrofungi [46]. Total genomic DNA was extracted from dried specimens using a cetyltrime-thylammonium bromide (CTAB) Rapid Plant Genome Extraction Kit (Aidlab Biotech-nologies Company, Ltd., Beijing, China) according to the manufacturer’s instructions with some modifications [47,48]. ITS were amplified using the primer pairs ITS5/ITS4 [49]. The PCR procedures for ITS followed Song et al. [50] and Sun et al. [51]. All PCR products were directly purified and sequenced at the Beijing Genomics Institute (BGI), China, with the same primers [52].

The obtained sequences were blasted in GenBank database (https://blast.ncbi.nlm.nih.gov, available accessed on 5 September 2024) to delimit species. Species identification was based on the morphological characterization and sequence comparison together. Fungal nomenclature followed the Index Fungorum (http://www.indexfungorum.org, available accessed on 13 September 2024).

### 2.4. Statistical Analysis

Four alpha diversity indexes, the Shannon diversity index [53], Richness index [54], Invsimpson diversity index [55], and Chao1 diversity index [55], were calculated using “vegan” package [55] to assess macrofungal alpha diversity. One-way analysis of variance (ANOVA) was used to test the difference in alpha diversity among the seven vegetation types. The *t*-test was used to test the difference in alpha diversity between two areas. Prior to analysis, normal distribution and variance homogeneity were checked using Shapiro–Wilk’s test and Bartlett’s test, respectively. Data were log-transformed to obtain a normal distribution if required. Principal coordinates analysis (PCoA) based on Bray–Curtis distances was used to evaluated beta diversity [56]. The permutational multivariate analysis of variance (PERMANOVA) was used to assess the difference in macrofungal community composition.

In addition, the Kruskal–Wallis (KW) test was used to assess the effect of different vegetation types on the number of species and genera. Analysis of similarity (ANOSIM) was used to calculate differences in macrofungal species composition and genera composition among the seven vegetation types. The quadratic regression was used to explore the relationship between species richness and elevation gradient. The “Venn” package was used to generate a Venn diagram. The high abundance of families and genera in the Dongling Mountains were shown using the “circlize” package. Statistical analysis and visualization of the data in this paper were conducted with R version 4.2.2.

## 3. Results

### 3.1. Macrofungal Composition

A total of 1379 macrofungal specimens were collected, and 311 species, belonging to two phyla, four classes, 18 orders, 74 families, and 147 genera were identified (Table 1). Basidiomycota is the dominant phylum, which contains one class, 13 orders, 65 families, 134 genera, and 284 species, while Ascomycota contains three classes, 5 orders, 9 families, 13 genera, and 27 species (Table 1). Agaricomycetes (284 species, 91.32%) and Pezizomycetes (21 species, 6.75%) were the most abundant classes. Agaricales (179 species, 57.56%) and Polyporales (50 species, 16.08%) were the most abundant orders. Among the 311 macrofungal species, there were 52 edible species, such as *Lactarius deliciosus* and *Pleurotus pulmonarius*; 38 medicinal species, such as *Ganoderma applanatum* and *Sanghuangporus baumii*; and 35 poisonous species, such as *Pholiota squarrosa* and *Leccinum scabrum*.

Among the identified species, there were nine dominant families (number of species ≥ 10 species) of macrofungi (Table 2). The Polyporaceae and Psathyrellaceae were the most diverse families. In addition, 65 families contained less than 10 species, accounting for 87.84% of the families and 59.16% of the identified species.

Among the identified species, there were 14 dominant genera (number of species ≥ 5 species) of macrofungi (Table 3). The Hebeloma and Mycena were the most diverse genera. In addition, 50 genera contained 2–4 species, accounting for 34.01% of the genera and 41.48% of the identified species; 83 genera contained only one species, accounting for 56.46% of the genera and 26.69% of the identified species.

### 3.2. Cumulative Abundance of Macrofungi in Seven Vegetation Types

**Figure 2:**
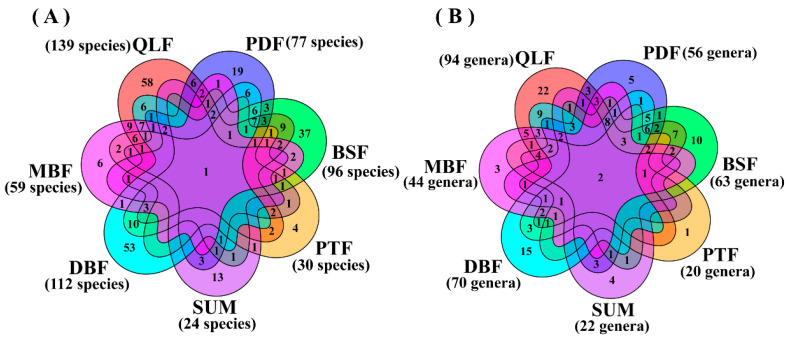
The shared genera and species were analyzed in seven vegetation types. (**A**) Shared species analysis in seven vegetation types. (**B**) Shared genera analysis in seven vegetation types. The numbers in parentheses are values of all observed macrofungal genera and species in each vegetation type studied (cumulative richness).

One species (*Marasmius siccus*) was shared in seven vegetation types. There were the fewest unique species (found only in one vegetation type) in PTF, and the most unique species in QLF (Figure 2A). A total of 58 unique species were found in QLF, such as *Xylaria primorskensis* and *Trechispora sinensis*; 53 unique species were found in DBF, such as *Xenasmatella vaga* and *Skeletocutis lepida*; 37 unique species were found in BSF, such as *Xerocomus ferrugineus* and *Stereum subtomentosum*; 19 unique species were found in PDF, such as *Scutellinia colensoi* and *Pluteus terricola*; 13 unique species were found in SUM, such as *Tubaria conspersa* and *Psilocybe atrobrunnea*; 6 unique species were found in MBF, such as *Dentocorticium ussuricum* and *Cyclocybe erebia*; 4 unique species were found in PTF, such as *Marasmius delectans* and *Diplomitoporus flavescens*. With the species richness increasing, the vegetation types in sequence are SUM, PTF, MBF, PDF, BSF, DBF, and QLF. The Kruskal–Wallis test (*p* > 0.05) indicated no significant effect of vegetation type on the number of macrofungal species. The ANOSIM (R = 0.811, *p* = 0.001) indicated significant differences in species composition across vegetation types. *Gymnopus* and *Marasmius* were shared in seven vegetation types. The most unique genera (found only in one vegetation type) in QLF and the fewest unique genera in PDF (Figure 2B). A total of 22 unique genera were found in QLF, such as *Xylaria*, *Echinoderma,* and *Artomyces*; 15 unique genera were found in DBF, such as *Mallocybe*, *Suillellus,* and *Paxillus*; 10 unique genera were found in BSF, such as *Lasiosphaeria*, *Simocybe,* and *Xerocomus*; 5 unique genera were found in PDF, such as *Pseudosperma*, *Pseudosperma,* and *Lentinus*. The four unique genera of SUM were *Clitopilus*, *Tubaria*, *Psilocybe,* and *Panaeolina*; the three unique genera of MBF were *Cyclocybe*, *Laetiporus,* and *Dentocorticium*; the only one unique genera of PTF was *Diplomitoporus*. With the number of genera increasing, the vegetation types in sequence are PTF, SUM, MBF, PDF, BSF, DBF, and QLF. The Kruskal–Wallis test (*p* > 0.05) indicated no significant effect of vegetation type on the number of genera. The ANOSIM (R = 0.7445, *p* = 0.001) indicated significant differences in genera composition across vegetation types.

**Figure 3:**
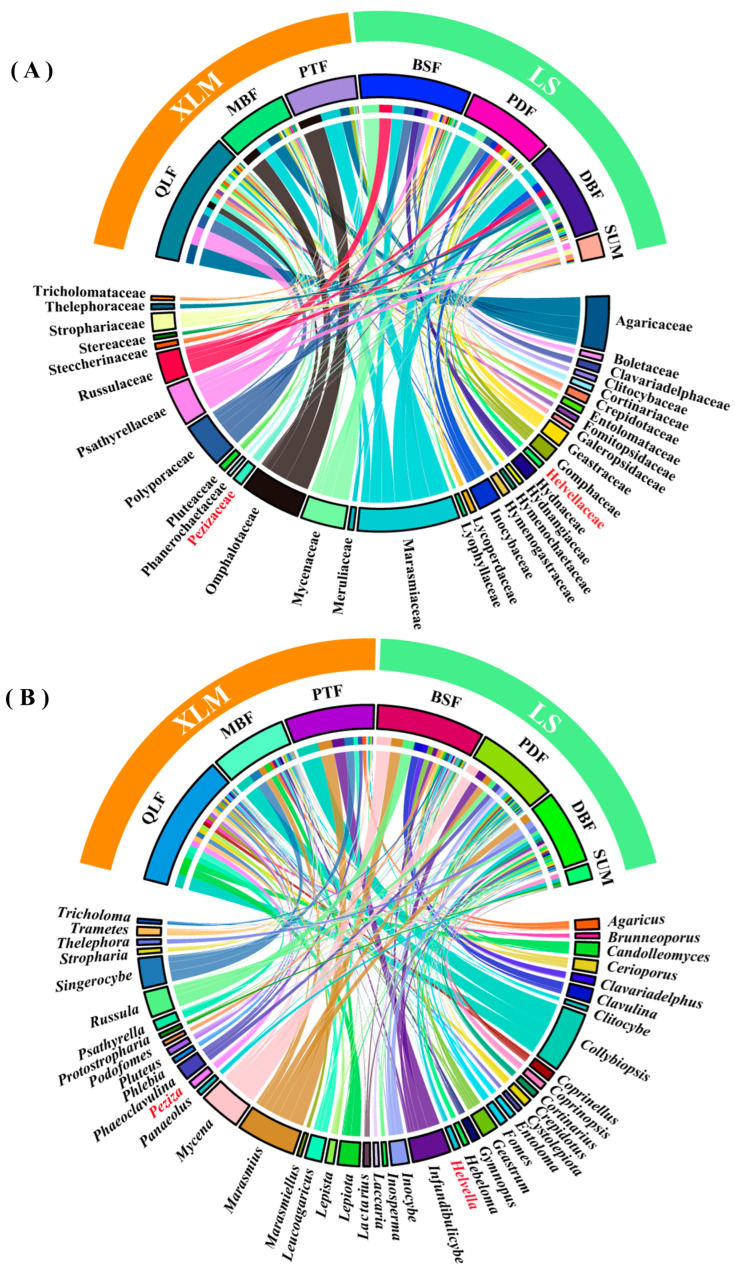
The Circos plot showed the main macrofungi in seven vegetation types. The abundance of families and genera with more than 10 were selected as basis for analysis. (**A**) Family abundance analysis in seven vegetation types. (**B**) Genera abundance analysis in seven vegetation types. The latin name in red are Ascomycota fungi, and those in black are Basidiomycota fungi.

Family abundance with more than 10 were selected for abundance analysis (Figure 3A). The QLF contained the most identified families among the seven vegetation types, totaling 54 families, while PTF contained the fewest families, totaling 12 families. Overall, the most reported one was Marasmiaceae. The dominant families in different vegetation types were not same, and the details are shown in Figure 3A.

Genera abundance with more than 10 were selected for abundance analysis (Figure 3B). The QLF contained the most genera among the seven vegetation types, totaling 94 genera, while PTF contained the fewest genera, totaling 20 genera. Overall, the most reported one was *Collybiopsis*. The dominant genera in different vegetation types were not the same, and the details are shown in Figure 3B.

### 3.3. Analysis of Macrofungal Diversity in Different Vegetation Types

**Figure 4:**
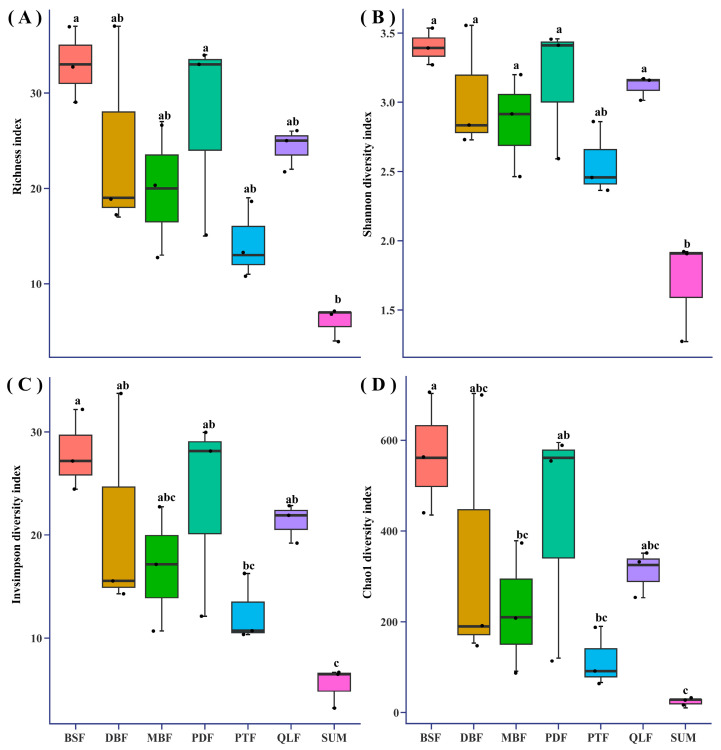
Alpha diversity analysis in seven vegetation types. (**A**) Richness index. (**B**) Shannon diversity index. (**C**) Invsimpson diversity index. (**D**) Chao1 diversity index. The center line of each box plot represents the median value of the data set, and the horizontal axis is the vegetation type. Different lowercase letters indicate significant difference among groups (*p* < 0.05).

The alpha diversity in seven vegetation types was assessed using four alpha diversity indexes. The four alpha diversity indexes showed that the alpha diversity in BSF and PDF was significantly higher than the other five vegetation types, while alpha diversity in SUM was significantly lower than the other six vegetation types (Figure 4). The four alpha diversity indexes in BSF and PDF were similar, and the alpha diversity index in DBF and MBF were similar (Figure 4).

**Figure 5:**
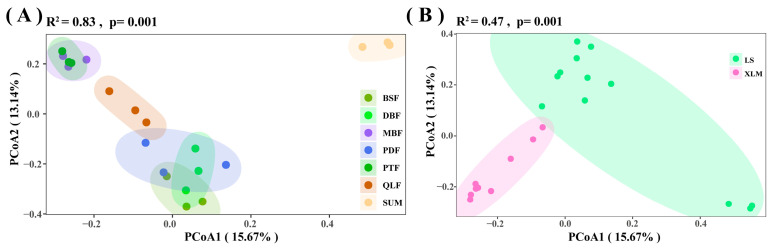
Principal coordinates analysis (PCoA) plot based on Bray–Curtis distance. (**A**) Seven vegetation types. (**B**) Two areas. Each point corresponds to a different sample, and different colors indicate different vegetation types or areas. The percentage of variation indicated on each axis corresponds to the proportion of the total variance explained by the projection.

The beta diversity (PCoA based on Bray–Curtis distances) and macrofungal community composition were used to assess the effect of different vegetation types on macrofungal community composition (Figure 5A). The PCoA1 and PCoA2 explained 15.67% and 13.14% of the total variation, respectively (Figure 5A). Figure 5A shows high similarity in the macrofungal community composition of BSF, DBF, and PDF, as well as PTF and MBF. The macrofungal community composition of SUM separated significantly from the other six vegetation types (PERMANOVA, R^2^ = 0.83, *p* = 0.001) (Figure 5A).

### 3.4. Analysis of Macrofungal Diversity in Two Areas

**Figure 6:**
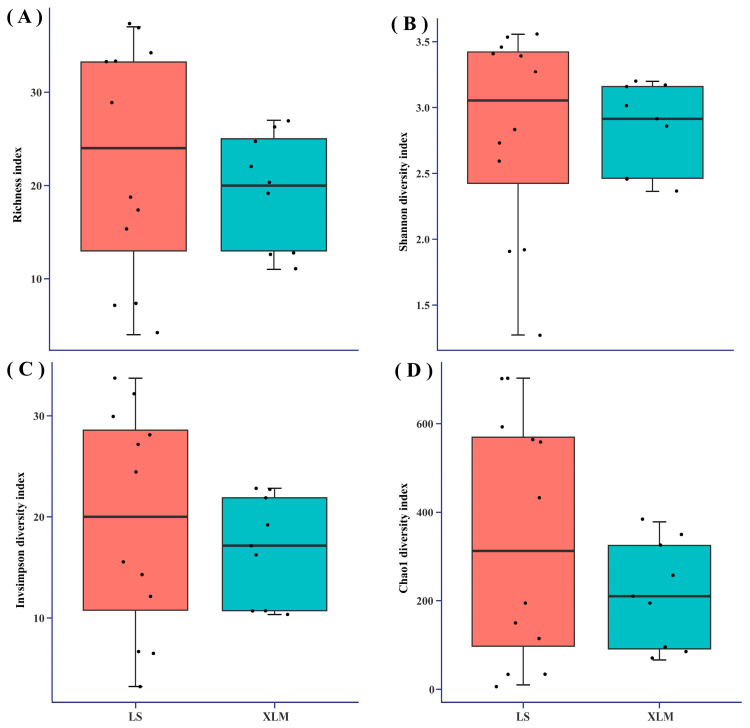
Alpha diversity analysis in two areas. (**A**) Richness index. (**B**) Shannon diversity index. (**C**) Invsimpson diversity index. (**D**) Chao1 diversity index. The center line of each box plot represents the median value of the data set, and the horizontal axis is area.

The Richness index, Shannon diversity index, Invsimpson diversity index, and Chao1 diversity index were used to assess the macrofungal alpha diversity in LS and XLM (Figure 6). The macrofungal alpha diversity of LS is higher than XLM indicating the macrofungal richness and evenness of LS were higher than XLM (Figure 6), but there was no significant difference (*p* > 0.05) between two areas.

The beta diversity (PCoA based on Bray–Curtis distances) and macrofungal community composition were used to assess the effect of different areas on macrofungal community composition (Figure 5B). The PCoA1 and PCoA2 explained 15.67% and 13.14% of the total variation, respectively (Figure 5B). Figure 5B indicates that the macrofungal community composition of the two areas were significantly different (PERMANOVA, R^2^ = 0.47, *p* = 0.001) (Figure 6B).

### 3.5. Macrofungal Species Distribution Along the Elevation Gradient

**Figure 7:**
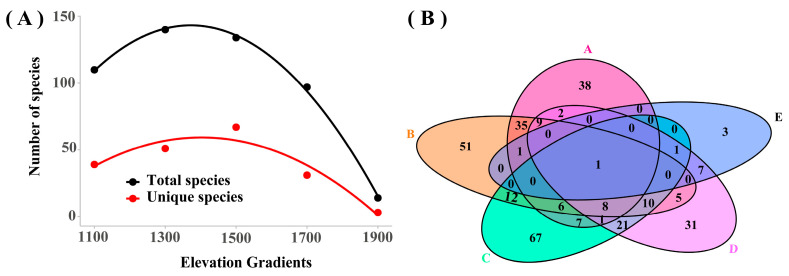
Variation in macrofungi along the elevation gradient. (**A**) The relationship between macrofungal species richness and elevation gradient. The horizontal axis is the elevation (the median value of each elevation gradient). (**B**) Shared species analysis in five elevation gradients. A: 1000–1200 m. B: 1200–1400 m. C: 1400–1600 m. D: 1600–1800 m. E: 1800–2000 m.

Figure 7A indicates that macrofungal species richness (total species in each elevation gradient) tended to increase and then decrease with increasing elevation (Figure 7A). The range of elevation from 1200 m to 1600 m presented abundant macrofungal species (Figure 7A); elevation gradient did not significantly (*p* > 0.05) affect macrofungal species richness.

*Marasmius siccus* was the only shared species in five elevation gradients. The elevation gradient C (1400–1600 m) contained the most unique species (67 species), while elevation gradient E (1800–2000 m) contained the fewest (3 species) (Figure 7B). Among the five elevation gradients, neighboring elevation gradients had more shared species (Figure 7B), suggesting that the macrofungal species composition of neighboring elevation gradients was more similar. Figure 7A indicates that the unique species (found only in one elevation gradient) tended to increase and then decrease with increasing elevation (Figure 7A), and elevation gradient did not significantly (*p* > 0.05) affect the number of unique species.

## 4. Discussion

The forests of the Dongling Mountains are well-preserved warm-temperate deciduous broad-leaved forests in China [38]. Our survey enriched the macrofungal diversity of the Dongling Mountains and Beijing, and more detailed data provided a better conservation and utilization of macrofungi in Beijing and warm-temperate deciduous broad-leaved forests.

More than 90% species of the macrofungi in the Dongling Mountains belonged to the Basidiomycota. In different forest ecosystems, the Basidiomycota is dominant had been documented by previous studies [11,57,58]. The reason is that most of the macrofungi species belong to Basidiomycota. While Ascomycota mainly contains microfungi, only a few taxa of Ascomycota are macrofungi. The families with high abundance in the Ascomycota are Pezizaceae and Helvellaceae, and the genera with high abundance are *Peziza* and *Helvella*. The Ascomycota and Basidiomycota contain the most saprophytic fungi, symbiotic fungi, and parasitic fungi, and play key roles in promoting the biogeochemical cycle and energy flow of ecosystems. Any comparison of macrofungal diversity found in different studies is difficult due to the size of the sampling area, specific geological and pedological characteristics, management intensity, and disturbance status [59,60]. In our study, the number of macrofungi was not the same in the three collection months; the most macrofungal species were collected in September, and the fewest were collected in July. The occurrence of macrofungi is not continuous and influenced by season [5,34]. Temperature and precipitation vary greatly in different months, and these factors affect the timing and development of fruit bodies [1], which explains the different number of macrofungi collected in different months. The Dongling Mountains suffered severe rainstorms in 2023–2024, which had affected the diversity of macrofungi. However, due to the lack of systematic surveys in the Dongling Mountains, it is difficult to judge the influence of rainstorms on our study.

### 4.1. Macrofungi and Vegetation Types

Macrofungal species composition and genus composition are different among the seven vegetation types in the Dongling Mountains (Figure 2 and Figure 3). In fact, each vegetation types contained a certain proportion of unique macrofungal species, accounting for 60.7% of the macrofungal species that were collected under only one vegetation type. Our results were similar to previous studies by Packham et al. [61] and O’hanlon et al. [62]. The Kruskal–Wallis test (*p* > 0.05) indicated no significant effect of vegetation type on the number of macrofungal species and genera. However, the alpha diversity analysis indicated there were significant differences in macrofungal diversity in the seven vegetation types (Figure 4). The reason for this contradiction is that the alpha diversity analysis considered not only the number of species but also the evenness of species distribution. This is because the Kruskal–Wallis test showed that vegetation type had no significant effect on the number of macrofungal species, which may suggest that the influence of vegetation type on macrofungal diversity is mainly because vegetation type affects the evenness of macrofungal distribution. Additionally, we used the number of species collected in the whole vegetation type in the Kruskal–Wallis test, but we used the correlation data in sample plots in the alpha diversity analysis, which might have generated the contradiction.

The PCoA based on the Bray–Crutis distance (R^2^ = 0.87, *p* = 0.001) indicated that there was a significant difference in macrofungal community composition under the seven vegetation types (Figure 5A). Additionally, ANOISM also showed significant differences in macrofungal community composition (species and genera) in different vegetation types. Previous studies indicated that macrofungi tend to present in specific forests, such as *Lactarius* [63,64], *Russula* [65,66], and *Cortinarius* [67,68], among which ectomycorrhizal fungi are mainly associated with *Quercus* and *Pinus*. Furthermore, the age of the forest affects the macrofungal diversity and community composition [69]; the different ages of the seven vegetation types may be the reason for the significant differences. Besides the different nutrition types, macrofungi are influenced by different factors; for example, saprophytic fungi are affected by soil pH, the mean volume of logs, and the decomposition stage of deadwood [24,25,26,27]. The conditions of the seven vegetation types were different, such as temperature, humidity, and amount of deadwood, which generated significant differences in macrofungal diversity and community composition.

### 4.2. Macrofungi in Different Areas and Elevations

The alpha diversity of LS was higher than XLM (Figure 6), but there was no significant difference (*p* > 0.05). Climatic conditions, especially precipitation and temperature [1], were key influencing factors of macrofungal diversity. The PCoA based on the Bray–Crutis distance showed significant differences (R^2^ = 0.47, *p* = 0.001) in macrofungal community composition between LS and XLM (Figure 5B). The different management of forests affected macrofungal community composition [70], while the management of LS and XLM were different, which may be the reason for the significant difference between the two areas. In addition, LS suffered serious rainstorms in the past few years. The macrofungal habitats in LS were destroyed, which may lead to a significant difference in macrofungal community composition between the two areas.

Macrofungal species richness and the number of unique species tended to increase and then decrease with increasing elevation (Figure 7A). Macrofungal species richness and composition in different elevation gradients were different (Figure 7), which were consistent with the results of previous studies [71,72]. Species richness peaked at an intermediate extent of human disturbance; communities with intermediate disturbance contained more species [73], which may explain the variation trend of species richness along the elevation. Microhabitat specialization was important for sporocarp formation and macrofungal species diversity [18], which was one of the main reasons why the elevation gradient did not significantly affect (*p* > 0.05) macrofungal species richness and the number of unique species, and was the reason why the neighboring elevation gradients of macrofungal species composition were more similar.

## 5. Conclusions

This study revealed the macrofungal diversity and community composition in different vegetation types, areas, and elevations of the Dongling Mountains. The results showed that vegetation type significantly affected macrofungal abundance and diversity, but the diversity between the two areas was not significant. Vegetation type and area significantly effected macrofungal community composition. Macrofungal species richness and the number of unique species tended to increase and then decrease with increasing elevation. The macrofungal species composition of neighboring elevation gradients was more similar. This study provided a theoretical basis for the conservation of macrofungal diversity and resources in the Beijing area of China.

## Figures and Tables

**Table 1:** Composition of the macrofungal flora in different vegetation types.

Phylum	Class	Order	Family	Genus	Vegetation Type
Ascomycota	Leotiomycetes	Helotiales	Gelatinodiscaceae	*Ascocoryne*	BSF
	Pezizomycetes	Incertae sedis	Tarzettaceae	*Tarzetta*	BSF, DBF, PDF
		Pezizales	Helvellaceae	*Helvella*	BSF, QLF, DBF, PDF, PTF, MBF
			Pezizaceae	*Adelphella*	DBF
				*Pachyphlodes*	DBF
				*Paragalactinia*	BSF, QLF, DBF, PDF
				*Peziza*	QLF, MBF
			Pyronemataceae	*Scutellinia*	QLF, PDF
			Sarcoscyphaceae	*Sarcoscypha*	QLF, MBF
	Sordariomycetes	Sordariales	Lasiosphaeriaceae	*Lasiosphaeria*	BSF
		Xylariales	Xylariaceae	*Xylaria*	QLF
		Hypocreales	Hypocreaceae	*Sepedonium*	DBF
				*Hypomyces*	QLF, DBF
Basidiomycota	Agaricomycetes	Agaricales	Agaricaceae	*Agaricus*	SUM, BSF, QLF, DBF, PTF, MBF
				*Cystolepiota*	QLF, PDF, PTF, MBF
				*Echinoderma*	QLF
				*Lepiota*	QLF, DBF, PDF, PTF, MBF
				*Leucoagaricus*	QLF, MBF, DBF, PTF
				*Leucocoprinus*	SUM, QLF, DBF, PDF, MBF
			Amanitaceae	*Amanita*	QLF, DBF, MBF
			Bolbitiaceae	*Conocybe*	SUM, PDF
				*Pholiotina*	QLF
			Clitocybaceae	*Clitocybe*	BSF, QLF, DBF, PDF, PTF, MBF
			Cortinariaceae	*Cortinarius*	BSF, DBF, QLF
			Crepidotaceae	*Crepidotus*	BSF, QLF, DBF, PDF, MBF
				*Simocybe*	BSF
			Entolomataceae	*Clitopilus*	SUM
				*Entoloma*	BSF, QLF, DBF, PDF, MBF
			Galeropsidaceae	*Panaeolina*	SUM
				*Panaeolus*	SUM, PDF
			Hydnangiaceae	*Laccaria*	QLF, PDF
			Hygrophoraceae	*Hygrocybe*	BSF, DBF
				*Hygrophorus*	BSF
			Hymenogastraceae	*Galerina*	QLF, MBF
				*Hebeloma*	BSF, QLF, DBF, PDF
				*Psilocybe*	SUM
			Incertae sedis	*Infundibulicybe*	BSF, QLF, DBF, PDF, PTF, MBF
				*Lepista*	BSF, QLF, DBF, PDF, MBF
				*Melanoleuca*	SUM, BSF, QLF, PDF, PTF, MBF
				*Ripartites*	QLF, PDF
				*Singerocybe*	BSF, QLF, PDF, PTF, MBF
			Inocybaceae	*Inocybe*	BSF, QLF, DBF, PDF, MBF
				*Inosperma*	BSF, DBF, PDF
				*Mallocybe*	DBF
				*Pseudosperma*	PDF
			Lycoperdaceae	*Lycoperdon*	SUM, BSF, QLF, DBF, PDF
			Lyophyllaceae	*Asterophora*	PDF, DBF
				*Calocybe*	QLF, DBF, PDF, MBF
			Macrocystidiaceae	*Macrocystidia*	QLF
			Marasmiaceae	*Marasmius*	SUM, BSF, PDF, DBF, MBF, PTF, QLF
			Mycenaceae	*Hemimycena*	PDF
				*Mycena*	BSF, QLF, DBF, PDF, PTF, MBF
			Omphalotaceae	*Collybiopsis*	QLF, PDF, PTF, MBF
				*Gymnopus*	SUM, BSF, QLF, DBF, PDF, PTF, MBF
				*Marasmiellus*	QLF
				*Rhodocollybia*	QLF, DBF
			Phyllotopsidaceae	*Phyllotopsis*	QLF, DBF, PDF
			Physalacriaceae	*Armillaria*	PDF, MBF
				*Flammulina*	QLF, MBF
			Pleurotaceae	*Pleurotus*	BSF, QLF, PDF
			Pluteaceae	*Pluteus*	BSF, QLF, PDF, PTF, MBF
				*Volvariella*	QLF
			Psathyrellaceae	*Candolleomyces*	BSF, QLF, DBF, MBF
				*Coprinellus*	SUM, BSF, QLF, DBF, PDF
				*Coprinopsis*	SUM, BSF, QLF
				*Parasola*	SUM, QLF, MBF
				*Psathyrella*	SUM, BSF, QLF, DBF, MBF
			Radulomycetaceae	*Radulomyces*	BSF, QLF
			Schizophyllaceae	*Schizophyllum*	SUM, QLF, DBF, PDF, MBF
			Strophariaceae	*Agrocybe*	SUM, QLF, PDF
				*Deconica*	SUM, BSF
				*Kuehneromyces*	BSF, DBF
				*Pholiota*	QLF
				*Protostropharia*	SUM, BSF, PDF
				*Stropharia*	QLF, PDF, PTF, MBF
			Tricholomataceae	*Tricholoma*	BSF, QLF, DBF, PDF
			Tubariaceae	*Cyclocybe*	MBF
				*Tubaria*	SUM
		Amylocorticiales	Amylocorticiaceae	*Plicaturopsis*	BSF, DBF, PDF
		Auriculariales	Auriculariaceae	*Tremellochaete*	QLF
		Boletales	Boletaceae	*Leccinum*	BSF, DBF
				*Suillellus*	DBF
				*Xerocomus*	BSF
			Hygrophoropsidaceae	*Leucogyrophana*	QLF
			Paxillaceae	*Paxillus*	DBF
			Suillaceae	*Suillus*	QLF
			Tapinellaceae	*Tapinella*	QLF
		Cantharellales	Hydnaceae	*Clavulina*	BSF, DBF, PDF
		Corticiales	Corticiaceae	*Lyomyces*	QLF, BSF
		Geastrales	Geastraceae	*Geastrum*	BSF, QLF, DBF, PDF, MBF
		Gomphales	Clavariadelphaceae	*Clavariadelphus*	BSF, DBF, PDF
			Gomphaceae	*Phaeoclavulina*	QLF, PDF, PTF, MBF
		Hymenochaetales	Hirschioporaceae	*Pallidohirschioporus*	BSF, PDF, QLF
			Hymenochaetaceae	*Hydnoporia*	BSF
				*Phellinus*	DBF
				*Phellinopsis*	QLF
				*Sanghuangporus*	DBF
			Hyphodontiaceae	*Hyphodontia*	BSF
			Oxyporaceae	*Oxyporus*	QLF, PDF
			Rickenellaceae	*Cotylidia*	QLF
		Polyporales	Climacocystaceae	*Diplomitoporus*	PTF
			Cerrenaceae	*Cerrena*	BSF, QLF
			Fomitopsidaceae	*Brunneoporus*	BSF, QLF, MBF
				*Fomitopsis*	QLF, DBF
			Hyphodermataceae	*Hyphoderma*	QLF
				*Mutatoderma*	QLF, DBF, MBF
			Incrustoporiacea	*Skeletocutis*	BSF, QLF, DBF
			Irpicaceae	*Byssomerulius*	DBF
				*Efibula*	QLF
				*Irpex*	BSF, QLF, MBF
				*Vitreoporus*	BSF, QLF
			Laetiporaceae	*Laetiporus*	MBF
			Meruliaceae	*Irpiciporus*	DBF
				*Phlebia*	QLF, MBF, DBF
			Phanerochaetaceae	*Bjerkandera*	QLF, DBF
				*Phanerochaete*	QLF
				*Phlebiopsis*	QLF, DBF
				*Porostereum*	QLF
			Polyporaceae	*Cerioporus*	BSF, QLF, DBF, PDF, MBF
				*Daedaleopsis*	BSF, QLF, PDF
				*Dentocorticium*	MBF
				*Fomes*	BSF, QLF, DBF, PDF
				*Ganoderma*	QLF, DBF
				*Lentinus*	PDF
				*Lopharia*	DBF
				*Panus*	SUM, PDF
				*Picipes*	BSF
				*Podofomes*	BSF, QLF
				*Polyporus*	QLF, DBF, MBF
				*Trametes*	BSF, QLF, DBF, PDF, PTF
			Postiaceae	*Cyanosporus*	QLF
			Steccherinaceae	*Antella*	BSF
				*Antrodiella*	QLF
				*Junghuhnia*	BSF, QLF
				*Steccherinum*	BSF, QLF, DBF, PTF
		Russulales	Auriscalpiaceae	*Artomyces*	QLF
			Bondarzewiaceae	*Lauriliella*	QLF
			Peniophoraceae	*Peniophora*	DBF
				*Scytinostroma*	DBF
			Russulaceae	*Lactarius*	BSF, QLF, DBF, PTF, MBF
				*Russula*	SUM, BSF, QLF, DBF, PDF
			Stereaceae	*Aleurodiscus*	QLF, DBF
				*Stereum*	BSF, PDF, DBF, MBF
			Xenasmataceae	*Xenasmatella*	DBF
		Thelephorales	Thelephoraceae	*Thelephora*	DBF
		Trechisporales	Hydnodontaceae	*Trechispora*	QLF

**Table 2:** Dominant families (≥10 species) of macrofuni in the Dongling Mountains.

Family	Number of Species	Percentage (%)
Psathyrellaceae	19	6.11%
Agaricaceae	17	5.47%
Polyporaceae	16	5.14%
Inocybaceae	15	4.82%
Russulaceae	14	4.50%
Hymenogastraceae	13	4.18%
Omphalotaceae	12	3.86%
Mycenaceae	11	3.54%
Strophariaceae	10	3.22%
Total	127	40.84%

**Table 3:** Dominant genera (≥5 species) of macrofungi in the Dongling Mountains.

Genus	Number of Species	Percentage (%)
*Hebeloma*	10	3.22%
*Mycena*	10	3.22%
*Cortinarius*	9	2.89%
*Russula*	9	2.89%
*Helvella*	8	2.57%
*Inocybe*	7	2.25%
*Agaricus*	6	1.93%
*Collybiopsis*	6	1.93%
*Coprinellus*	6	1.93%
*Entoloma*	6	1.93%
*Inosperma*	6	1.93%
*Pluteus*	6	1.93%
*Lactarius*	5	1.61%
*Psathyrella*	5	1.61%
Total	99	31.83%

## Data Availability

The original contributions presented in this study are included in the article. Further inquiries can be directed to the corresponding authors.
